# Case report: Primary hepatocellular carcinoma with portal vein tumor thrombus characterized by active tumor immune microenvironment achieving a complete response following treatment of combined immunotherapy

**DOI:** 10.3389/fimmu.2022.999763

**Published:** 2022-08-31

**Authors:** Kaihang Zhong, Yuyan Xu, Yuan Cheng, Yaohong Wen, Lei Cai, Guolin He, Huakun Huang, Shunjun Fu, Xuefeng Zhong, Yating Zheng, Tingting Chen, Mengli Huang, Mingxin Pan

**Affiliations:** ^1^ Department of Hepatobiliary Surgery II, General Surgery Center, Zhujiang Hospital, Southern Medical University, Guangzhou, China; ^2^ The Medical Department, 3D Medicines, Inc., Shanghai, China

**Keywords:** hepatocellular carcinoma (HCC), portal vein tumor thrombus (PVTT), tumor immune microenvironment (TIME), tertiary lymphoid structure (TLS), combined immunotherapy

## Abstract

Portal vein tumor thrombus (PVTT) is a frequent complication in hepatocellular carcinoma (HCC). HCC patients with PVTT have the characteristics of less treatment tolerance and poor prognosis. Immunotherapy, especially combined immunotherapy, has been successfully used in advanced HCC. However, there are no recognized universally indicators that can predict response or resistance to immunotherapy for HCC. Herein, we reported a 58-year-old HCC patient with PVTT, cirrhosis and chronic viral hepatitis, who achieved complete response (CR) after combined immunotherapy (camrelizumab combined with sorafenib or regorafenib), according to his high enrichment of tumor-infiltrating immune cells and tertiary lymphoid structure (TLS). In this case, we revealed the characteristics of the baseline tumor immune microenvironment (TIME) in a HCC patient who responded well to combined immunotherapy, suggesting that TIME can be used to assist in clinical decision making of immunotherapy for HCC.

## Introduction

Hepatocellular carcinoma (HCC) is a common gastrointestinal malignancy. Due to its biological and anatomical characteristics, HCC tumor cells easily invade the intrahepatic portal vein system and form portal vein tumor thrombus (PVTT). The incidence of HCC accompanied by PVTT was 44%~62.2% (1). HCC patients with PVTT usually have the characteristics of rapid disease progression, difficult treatment and poor prognosis, the median survival period without any therapies for only 2.7 months (1). However, there is no international consensus on the diagnosis and treatment criteria for HCC with PVTT, which brings great difficulties in therapeutic schemes selection and therapeutic effects prediction.

In recent years, hepatic resection, regional interventional therapy, radiotherapy, molecule targeted therapy and comprehensive therapies have been widely used, which is of great significance to the quality of life and prolonged the survival time in partial patients (2). Western clinicians regard HCC patients with PVTT as advanced patients that are difficult to operate. Hepatic resection with or without thrombectomy began to be a widespread practice for HCC patients with PVTT in Asian liver centers. A large retrospective study in Japan found that the median overall survival (OS) was significantly longer in HCC patients with PVTT who underwent hepatectomy than in those who did not (2.87 years vs. 1.10 years) (3). Transarterial chemoembolization (TACE) is a standard treatment for unresectable HCC patients, but TACE showed less effective and safe than hepatic resection in HCC patients with PVTT (4). The current Barcelona Clinic Liver Cancer (BCLC) staging system categorizes HCC with PVTT as the advanced stage so that sorafenib or lenvatinib is generally recommended as first-line treatment (1). Although sorafenib has become the first-line systemic agent for advanced HCC, the survival benefit of patients is still limited, with a median OS improvement of about 2 months (5). Lenvatinib, cabozantinib and ramucirumab are promising options for advanced HCC, but their impacts on clinical outcomes in HCC patients with PVTT are unclear (1). Although the evidence of the efficacy of systemic therapy for advanced HCC is gradually expanding, the treatment guidance data for the subgroup of HCC patients with PVTT are still limited.

Immune checkpoint inhibitors (ICIs) have been successfully used in tumor therapy, among which inhibitors of programmed death ligand-1 (PD-L1) and its receptor programmed death-1 (PD-1) are commonly administered for HCC as alone or in combination with other treatments (6). The IMbrave150 trial showed that atezolizumab plus bevacizumab improved both OS and progression-free survival (PFS) compared with sorafenib so that this combined therapy has become the preferred first-line treatment of advanced HCC patients in clinical guidelines (7). Although ICIs has played a good effect in HCC, the overall objective response rate is still less than 40%, indicating that some patients still cannot benefit from it (6). Previous evidence suggests that HCC characterized by immune cell infiltrates and expression of immune checkpoint molecules may be good candidates for immunotherapy, whereas HCC showing intra-tumor T cells deficiency may be insensitive to ICIs (8). But there are no recognized and widely used indicators that can be used to predict response or resistance to HCC immunotherapy in clinical guidelines.

Here, we reported a HCC patient with PVTT, cirrhosis and chronic viral hepatitis, achieved CR and obtained significantly improved PFS following treatment with a PD-1 inhibitor combined with tyrosine kinase inhibitors (TKIs) as postoperative adjuvant therapy based on multiplex immunofluorescence (mIF) test results of tumor immune microenvironment (TIME).

## Case presentation

A 59-year-old Chinese male patient was admitted to our hospital on October 11, 2018 due to founding right hepatic occupying lesions for one week. He was a workman with 163 cm in height and 62 kg in weight, and had a history of chronic viral hepatitis B for more than 20 years with no previous antiviral therapy to control it.

**Figure 1:**
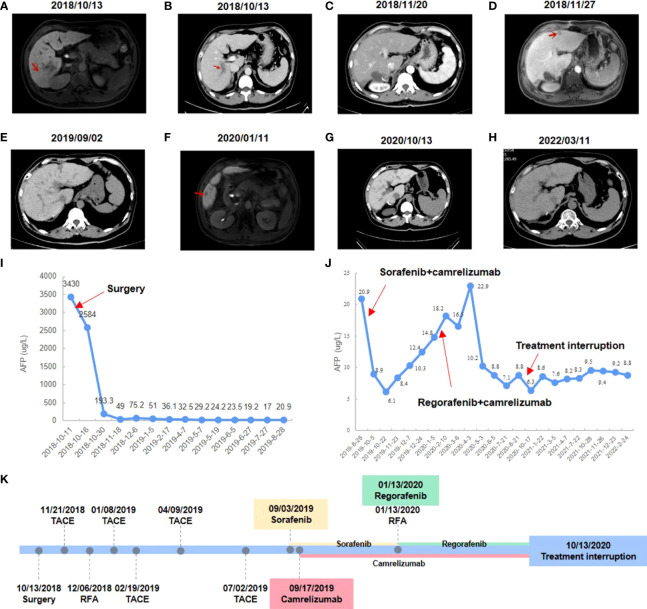
Changes in imaging and serum AFP level during clinical treatment. **(A)** Preoperative abdominal-enhanced CT revealed a mass in the right posterior lobe of the liver. **(B)** Preoperative CT showed tumor thrombus in the portal vein. **(C)** Postoperative abdominal-enhanced CT showed no obvious cancer foci. **(D)** MRI showed an enlarged nodule in segment II of the liver. **(E)** Enhanced CT showed no obvious recurrence of cancer. **(F)** MRI showed enlarged liver lesions in segment V. **(G)** Enhanced CT showed a CR to regorafenib and camrelizumab. **(H)** The latest enhanced CT showed no signs of recurrence. **(I)** AFP level changes during postoperative interventional treatment. **(J)** AFP level changes during camrelizumab combined with sorafenib or regorafenib. **(K)** Timeline of treatment process.

Laboratory examinations of the patient before therapy revealed alpha fetoprotein (AFP; a serum marker often used in the diagnosis and therapeutic efficacy monitoring of HCC) was 3430 μg/L. Abdominal B-ultrasound, enhanced computed tomography (CT), and magnetic resonance imaging (MRI) were performed and the patient was diagnosed with primary HCC with PVTT, cirrhosis and chronic viral hepatitis (**Figures 1A, B**). All the examination results suggested that the patient’s China Liver Cancer stage was stage IIIa, BCLC stage was stage C, and Cheng’s Classification was Type II. The patient’s liver function Child-pugh classification was grade A and Eastern Cooperative Oncology Group performance status score was grade 0.

Antiviral therapy (entecavir 0.5 mg/dose, 1 time/day, orally) combined with antitumor therapy (sophora granules, 20 g/dose, 3 times/day, orally) has been used since October 13, 2018. The patient underwent a right posterior lobe resection of the liver, portal vein dissection for embolization, and cholecystectomy on October 18, 2018. The patient had no significant postoperative complications. The patient’s AFP was rechecked one month after surgery and the result was 49 μg/L, and no obvious cancer foci were seen on enhanced CT examination (**Figure 1C**). TACE was performed on November 21, 2018, and adjuvant perfusion chemotherapy was used after intervention with the regimen of mFOLFOX6 for arterial perfusion: oxaliplatin 100 mg/m ^2,^ calcium folinic acid 400 mg/m ^2,^ and fluorouracil 2400 mg/m ^2.^ The patient underwent MRI on November 27, 2018, and the result showed an enlarged nodule in segment II of the liver, which was considered malignant (**Figure 1D**). B-ultrasound was performed on November 30, 2018 and suggested an enlarged nodule in the left outer lobe of the liver with a size of about 12×9 mm. On December 6, 2018, the patient’s AFP was rechecked and the result was 75.2 μg/L, and he underwent radiofrequency ablation (RFA) of liver tumors with image fusion magnetic navigation technology for suspicious lesions in segment II of the left lateral lobe. The patient underwent TACE on January 8, 2019, February 19, 2019, April 9, 2019, and July 2, 2019 respectively. Adjuvant infusion chemotherapy was performed after intervention every time, and the regimen was not changed. On August 28, 2019, the patient’s AFP was rechecked and the result was 20.9 μg/L. Over the past few months, the patient has monitored the changes in AFP monthly and performed the enhanced CT examination. Enhanced CT showed no obvious recurrence of cancer, but AFP results were abnormally increased (**Figures 1E, I**).

**Figure 2:**
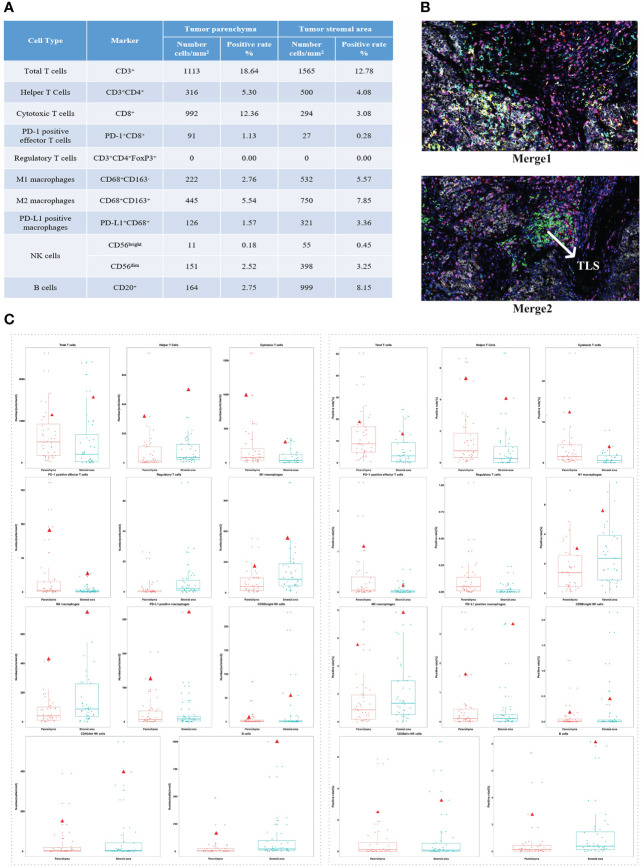
The patient’s immune cell test results and distribution in the total HCC population test data. **(A)** The test results of immune cells constituting tumor immune microenvironment. **(B)** The expression levels of all markers in tissue slides. Merge 1: PD-1 (green), PD-L1 (yellow), CD8 (pink), CD68 (cyan), CD163 (red). Merge 2: CD3(pink), CD4 (red), CD20 (green), CD56 (cyan), FoxP3 (yellow). **(C)** Distribution of the patient’s test results in the total HCC population test data (total: 40 patients). Left: the number of immune cells. Right: the positive rate of immune cells. The red triangle represents the patient’s test results.

After having obtained the consent of the patient and his family, FFPE slides of the liver tumor tissue were sent to conduct mIF staining using the Akoya OPAL Polaris 7-Color Automation IHC kit. TIME related markers, including PD-1, PD-L1, CD8, CD68, CD163, CD3, CD4, CD20, CD56 and FoxP3, were examined. Results showed that PD-L1 expression was positive (Ventana SP263; Tumor cell Proportion Score = 5%, Immune cell Proportion Score = 0%). Compared with the immune cell test data of the total HCC population, the test result of this patient was much higher than the average value of them, except for regulatory T cells (**Figures 2A, C**). It suggests that except for regulatory T cells, all of immune cells were enriched in both tumor parenchyma and tumor stromal area, especially cytotoxic T cells, M2 macrophages and CD56dim natural killer (NK) cells. Moreover, mIF staining of CD3 and CD20 reveals the high density of B cells and the presence of TLS in the HCC tumor (0.21/mm2; 11503.03 µm2/mm2; **Figure 2B**). All of these suggested that the patient may be sensitive to ICIs. Considering the patient’s physical score, mIF test results, drug availability and so on, combined immunotherapy was started in September 2019. On September 3, 2019, the patient initiated to treat with sorafenib tosylate (Nexavar, 0.4g/dose, 2 times/day, orally). And on September 17, 2019, the patient initiated to treat with camrelizumab (a PD-1 inhibitor, 200mg/dose, intravenous infusion every two weeks). On October 5, 2019, the patient’s AFP level was 8.9 μg/L. Since September 17, 2019, camrelizumab has been used once every two weeks. During this period, the patient did not complain any discomfort and no obvious complications were observed. The patient took sorafenib from September 3, 2019 to January 13, 2020, and no obvious complications occurred during the period. The liver lesion in the V segment was enlarged and the AFP began to increase (**Figures 1F, J**), so the TKIs drug was changed from sorafenib to regorafenib (0.16g/dose, once/day, orally). At the same time, CT-guided RFA of liver tumors was performed for suspicious tumor in the V segment of the right anterior lobe. The changes of AFP level after the combination of camrelizumab and TKIs were shown in the **Figure 1J**, showing AFP decreased to normal from June 2020 and then stabilized. The treatment was discontinued after a comprehensive evaluation in October 13, 2020 (**Figures 1G, K**). After that, only antiviral therapy was given with regular follow-up.

The patient underwent enhanced CT examination on March 11, 2022, and no signs of tumor recurrence were found (**Figure 1H**). AFP was monitored during the same period and remained at normal levels. According to the modified Response Evaluation Criteria in Solid Tumors, the patient achieved a CR based on our comprehensive evaluation and the PFS was more than 31 months at the last calculation. In the future, the changes of AFP level will be monitored every 3 months, and the enhanced CT examination will be performed every 6 months to monitor whether the tumor relapses.

## Discussion

According to the previous Chinese guidelines and treatment experience, the main treatment for HCC patients with PVTT is surgical resection, followed by adjuvant therapy such as interventional therapy, antiviral therapy, and huaier granules (1). In addition, when serological tumor markers are abnormal but tumor formation cannot be detected by imaging, the options for therapy are often limited. Here, we presented a HCC patient with PVTT, cirrhosis and chronic viral hepatitis, who had disease recurrence after surgery and then received multiple interventional therapies, but his serum APF remained abnormally high. The patient was subsequently treated with camrelizumab combined with TKIs (sorafenib or regorafenib) and achieved CR after 13 months of treatment which has been sustained until now, giving a PFS of over 31 months at the last calculation.

Sorafenib or lenvatinib is recommended as the first-line TKIs therapy in HCC with PVTT currently (1). Regorafenib is often used as the second-line treatment for advanced HCC patients when sorafenib or lenvatinib treatment has failed (9). They all belong to vascular endothelial growth factor receptor associated multi-target TKIs. However, previous trials demonstrated that anti-angiogenesis TKIs monotherapy showed unsatisfied effect in advanced HCC survival. There are several studies which have shown that combination of TKIs and ICIs can significantly improve the survival benefit of advanced HCC patients (7, 10), so that combination therapy has emerged as a recent trend in advanced HCC treatment. These studies also indicated that not all HCC patients can benefit from combination therapy, one of the reasons may be that some patients have primary immune resistance. But there are no recognized indicators that can predict response or resistance to ICIs in guidelines, which brings great difficulties to make clinical decision. In this case, TIME related markers test results of the patient helped us to choose TKIs combined with ICIs as a priority.

The TIME is a highly heterogeneous microecosystem composed of tumor cells and their surrounding environment. Predicting the response of ICIs based on the characteristics of tumor immune infiltrates is a critical step to improve the success rate of ICIs treatment (9), especially for HCC patients. The TIME test results of this patient showed that all of immune cells, except for regulatory T cells, were enriched and PD-L1 expression was positive. The Checkmate-040 trial showed that compared with tumor PD-L1 negative patients, PD-L1 positive (≥1%) patients had a higher median OS after receiving nivolumab (11). And increased CD3 and CD8 showed a trend towards improved OS (11). In addition, the tumor-infiltrating leukocytes (mainly CD8+ cytotoxic T cells) and PD-L1 expression levels in the TIME were combined to search for potential immunotherapy sensitive candidates and ICIs are most likely to be effective only when TILs and PD-L1 are present together (12). Besides cytotoxic T cells, other immune cell subsets may also contribute to anti-tumor immunity, such as tumor-associated macrophages (TAMs) and NK cells. TAMs can be divided into different phenotypes, including M1 macrophages and M2 macrophages (13). In a phase 1b study of sintilimab (a PD-1 inhibitor) plus a bevacizumab biosimilar in advanced HCC, the high infiltration of M1 macrophages (≥118.1 cells/mm^2^) were found to be significantly correlated with a better clinical benefit and prolonged survival (13). Of note, we observed the presence of TLS, which is lymphoid-like aggregates forming in non-lymphoid tissues during chronic inflammation and tumor progression. In 2020, the correlation between TLS and immunotherapy has been reported successively in melanoma and sarcoma, which all showed that the formation of TLS was connected with the better response to ICIs treatment (14–16). In HCC, TLS has been reported to correlative with favorable clinical outcomes (17), but there is less evidence on the relationship between TLS and immune efficacy. Here, we reported the presence of TLS detected at baseline in a HCC patient with PVTT who had a survival benefit from camrelizumab combined with TKIs, suggesting that TLS may be used as a potential indicator to predict immunotherapy response.

In this case, TIME related markers detection were performed using the liver tumor tissue obtained from surgical excision, rather than the tissue sample obtained after interventional treatment. We performed RFA or TACE on newly discovered liver tumor lesions from imaging after surgery, which may have an impact on the efficacy evaluation of camrelizumab combined with TKIs. However, we believe that resisting tumor growth requires comprehensive treatment. Regardless of any treatment means, the benefit of patients is always the first priority. According to our clinical experience, the best treatment is surgery or RFA when tumor lesions are found through imaging examination. After postoperative TACE or RFA therapy, the enhanced CT of this patient showed no obvious tumor lesions, but his AFP results were abnormally increased which suggested the presence of minimal residual disease. In this situation, the contribution of camrelizumab combined with TKIs is undoubtedly the most prominent. Although several clinical studies about combination therapy for HCC have been reported, the combination of drugs is still limited. Considering the presence of PVTT in the patient, we selected camrelizumab combined with sorafenib firstly, but this combination did not make AFP to return to normal. In the RESORCE trial, regorafenib increased survival as a second-line therapy in HCC patients progressing on sorafenib and reduced hazard of death for patients with macrovascular invasion (18). Therefore, regorafenib was used to replace sorafenib to combine with camrelizumab and we found that regorafenib may be better than sorafenib in activating the immunotherapy response.

To sum up, this report described an advanced HCC with PVTT who was considered to be at high risk of postoperative recurrence due to the repeating rise of AFP after TACE/RFA adjuvant therapy, but AFP was reduced and PFS increased significantly after following treatment with a PD-1 inhibitor combined with TKIs as adjuvant therapy based on the TIME feature. In the absence of sufficient evidence to guide the treatment of HCC patients with PVTT, we tried different combinations of immunotherapy and TKIs therapy outside the guidelines and found that camrelizumab combined with regorafenib was effective, which provided a basis for systemic therapy of HCC patients with PVTT. Furthermore, to our knowledge, this is the first report that described the tumor infiltration immune cell atlas of a HCC patient with PVTT who can benefit from combined immunotherapy and provided a theoretical foundation for the continuation of immunotherapy in HCC patients.

## Data availability statement

The original contributions presented in the study are included in the article/supplementary material. Further inquiries can be directed to the corresponding author.

## Ethics statement

Written informed consent was obtained from the individual(s) for the publication of any potentially identifiable images or data included in this article.

## Author contributions

KZ, YX, YC, and MP were involved in the interpretation of the clinical data, the conception and the drafting of the manuscript and participated in the management of the patient. YW, LC, GH, HH, and SF were involved in the collation of clinical data and radiological images. XZ and YZ were involved in manuscript editing and performed immune cell data analysis. TC and MH were involved in the manuscript revision. All authors contributed to the article and approved the submitted version.

## Funding

This work was supported by fund from the National Natural Science Foundation of China (no.82072627) and Guangdong Basic and Applied Basic Research Foundation of China (no. 2021B1515230011).

## Conflict of interest

Authors XZ, YZ, TC, and MH were employed by 3D Medicines, Inc.

The remaining authors declare that the research was conducted in the absence of any commercial or financial relationships that could be construed as a potential conflict of interest.

## Publisher’s note

All claims expressed in this article are solely those of the authors and do not necessarily represent those of their affiliated organizations, or those of the publisher, the editors and the reviewers. Any product that may be evaluated in this article, or claim that may be made by its manufacturer, is not guaranteed or endorsed by the publisher.
